# ^99^Tc-MDP maintains bone mineral density for postmenopausal differentiated thyroid cancer patients with osteopenia under thyroid-stimulating hormone suppression

**DOI:** 10.3389/fendo.2025.1657617

**Published:** 2025-10-02

**Authors:** Yuanfang Zhang, Yingqiu Wang, Donghua Sun, Chao Ma

**Affiliations:** ^1^ Department of Nuclear Medicine and Neurology, Tenth People’s Hospital of Tongji University, Shanghai, China; ^2^ Department of Nuclear Medicine, Yangpu Hospital of Tongji University, Shanghai, China

**Keywords:** differentiated thyroid cancer, ^99^Tc-MDP, thyroid stimulating hormone suppression, bone mineral density, osteopenia

## Abstract

**Background:**

The main determinant of skeletal fragility in postmenopausal patients with differentiated thyroid cancer (DTC) and osteopenia is thyroid-stimulating hormone (TSH) suppressive therapy. Evidence for the use of bisphosphonates, including technetium-99 methylene diphosphonate (^99^Tc-MDP), in this clinical setting remains limited.

**Objective:**

To investigate the effects of ^99^Tc-MDP on osteopenia (T-score <-1.0≥-2.5 SD for the lumbar spine by DXA) in postmenopausal women with DTC under TSH suppression therapy compared with routine calcium/vitamin D supplementation.

**Methods:**

A total of 102 postmenopausal patients with DTC and osteopenia under TSH suppression therapy were enrolled in this open-label, prospective study. Patients were divided into two groups: calcium/vitamin D supplements (group_Ca_) and calcium/vitamin D plus ^99^Tc-MDP (group_mdp_) groups. Lumbar spine bone mineral density (BMD) by DXA was measured before and 12 months after treatment. Bone turnover markers were evaluated at baseline, 6 months, and 12 months.

**Results:**

The combined ^99^Tc-MDP treatment significantly increased the mean percentage change of lumbar BMD at month 12 compared with group_Ca_ (t=2.156, p=0.035). A significant decrease in BMD of the lumber spine from 0.9148 ± 0.08 to 0.8726 ± 0.08 (t=3.81, p=0.001) at month 12 was observed in group_Ca_. The mean percentage change from baseline in the levels of serum β-isomer of C-terminal telopeptide of type I collagen (β-CTX), procollagen type 1 N-terminal propeptide (P1NP) showed that ^99^Tc-MDP combined treatment significantly increased PINP at month 6 (t=2.37, p = 0.02) and 12 (t=2.224, p = 0.029), and significantly decreased β-CTX at month 12 (t=-2.746, p = 0.008) compared with group_Ca_. No severe adverse events were reported in either group.

**Conclusions:**

^99^Tc-MDP is safe and could maintain lumbar BMD in postmenopausal women with DTC and osteopenia under TSH suppression therapy during a 1-year follow-up. Calcium/vitamin D supplementation alone did not effectively prevent bone loss in these patients.

**Trial registration number:**

ChiCTR2200064170

## Introduction

Differentiated thyroid cancer (DTC) has become the most common endocrine malignancy. Papillary and follicular thyroid cancer are the two main histological types. According to the American Thyroid Association (ATA) and Chinese Thyroid Association (CTA), most patients with DTC undergo total or near-total thyroidectomy, radioiodine ablation, and thyroid-stimulating hormone (TSH) suppression (1, 2). TSH suppression therapy is necessary in DTC because tumor cells express TSH receptors on cell membranes and respond to TSH stimulation by increasing the expression of several proteins and the rate of cell growth (3, 4). However, our previous study found that excessive intake of levothyroxine (L-T_4_) contributed to a negative balance of bone formation and resorption resulting in bone loss (5). Postmenopausal women with DTC receiving TSH suppression therapy are particularly vulnerable to osteopenia (OP) (6–11).

^99^Tc-methylene diphosphonate (^99^Tc-MDP), a chemical compound of technetium-99 conjugated with methylene diphosphonate ([^99^Tc-MDP], or Yunke, Chengdu Yunke Pharmaceutical Co., Ltd., Chengdu, Sichuan, China), is an anti–bone destruction drug patented in China. It has been widely used with good efficacy for the treatment of rheumatoid arthritis (RA) (patent No. ZL94113006.1) (12) and osteoporosis (patent No. ZL00100083.7) in China since 2000. Therefore, it is mainly indicated for RA and osteoporosis as described in its drug instructions. Our previous study showed that ^99^Tc-MDP was as efficacious as alendronate in improving lumbar bone mineral density (BMD) in DTC patients with osteoporosis under TSH suppression therapy (13). In clinical practice, we routinely encourage postmenopausal DTC patients under TSH suppression therapy to take oral calcium/vitamin D supplements. The BMD maintenance effect of the ^99^Tc-MDP was supposed in the current clinical study.

## Patients and methods

### Study design

This was part of an open-label, non-randomized clinical study (ChiCTR2200064170).

### Primary endpoint

Lumbar spine bone mineral density (BMD) before and 12 months after treatment.

### Secondary endpoints

Bone turnover markers, including serum β--isomerized of C-terminal telopeptide of type I collagen (β-CTX) and procollagen type 1 N-terminal propeptide (P1NP), and adverse events were evaluated at baseline, 6 months, and 12 months after treatment.

### Setting and participants

Postmenopausal DTC patients with OP under TSH suppression therapy from March 2022 to December 2022 were enrolled if they fulfilled all the following criteria. (1) They were pathologically diagnosed with DTC, including papillary or follicular carcinoma; (2) received a near-total thyroidectomy and radioiodine treatment; (3) bone mineral density (BMD) of the lumbar spine was tested by dual-energy X-ray absorptiometry (DXA) at baseline and at 12 months; (4) TSH suppression therapy was defined as a TSH level between 0.1–0.5 μIU/mL and had lasted for at least 1 year before the study; (5) they had osteopenia, namely, a T-score <-1.0≥-2.5 SD for the lumbar spine. They were followed up for at least 1 year.

We excluded patients who met the following criteria: (1) they had received medications for OP before TSH suppression treatment; (2) had secondary OP owing to parathyroid gland or kidney disease; (3) had severe liver or kidney disease; (4) had long-term use of an immunosuppressive agent, estrogen, or estrogen receptor modulators.

This study was approved by the Institutional Review Board of Research Ethics of Shanghai Tenth People’s Hospital. All patients were fully informed about their treatment and consented to participate in the clinical trial.

### TSH suppression therapy

TSH suppression treatment was based on the risk stratification of DTC using L-thyroxine (L-T_4)_ as recommended (1, 2): (1) For patients with persistent disease, TSH suppression below 0.1 μIU/mL is recommended. (2) For patients free of disease but originally presenting with high-risk disease, TSH suppression from 0.1 to 0.5 μIU/mL is recommended. (3) For patients with low risk of recurrence, TSH suppression from 0.3 to 2 μIU/mL is recommended. The dose of L-T_4_ was maintained stable during the study period. Free T_3_, free T_4_, and TSH were measured using a time-resolved immunofluorometric assay (Anytest, Sym-Bio Lifescience Co., Ltd., Shanghai, China).

### Treatment protocol

Patients were divided into two groups: calcium/vitamin D supplements (group_Ca_) and calcium/vitamin D combined ^99^Tc-MDP (group_mdp_) groups.

1. group_Ca_: Calcium carbonate 1200mg and vitamin D (afalciferol) 0.25μg once a day were orally administered.
2. group_mdp_: Calcium carbonate 1200mg and vitamin D (afalciferol) 0.25μg once a day were orally administered. In addition, ^99^Tc-MDP 10 mg was intravenously administered weekly for 10 weeks, then once every 2 weeks for 22 weeks, and monthly for another 5 months.

### BMD in spine lumbar

DXA (v.13.20; enCORETM 2009, GE Healthcare) was used to measure BMD at the L_1–4_ vertebral regions. Precision errors, established with a local normal population, were less than 1.5% for all locations at baseline and at 12 months.

### Serum bone turnover markers

Serum β-CTX, P1NP, and bone alkaline phosphatase (ALP) were determined by enzyme-linked immunosorbent assay (Modular E170, Hoffmann-La Roche, Basel, Switzerland) with intra- and inter-assay coefficients of variation (CVs) of 2.7% and 3.4%, respectively.

### Adverse reaction

Laboratory assays for routine blood tests, liver, and renal function were measured at baseline and 12 months. A treating physician reviewed the clinical results and any discomfort at each visit.

### Study size

The predetermined primary endpoint was the difference in the change in BMD of the lumbar spine between the two groups. Group samples of 46 and 46 achieved 80% power to detect superiority using a one-sided two-sample t-test. The margin of superiority was 0.036. The significance level (alpha) of the one-sided test was 0.025. The standard deviations of the two groups were 0.05 and 0.07, respectively. Considering a 10% loss to follow-up, the group sample size was 51 patients per group.

### Statistical methods

Continuous data are expressed as the mean ± standard deviation. The independent-sample t-test and Fisher’s exact chi-square test in SPSS 22 were used to compare baseline information and clinical characteristics within groups, and to determine differences in BMD values between baseline and 12 months after treatment. Differences in bone turnover markers and other laboratory results were determined using a two-sided t-test.

## Results

### Clinical characteristics

**Figure 1:**
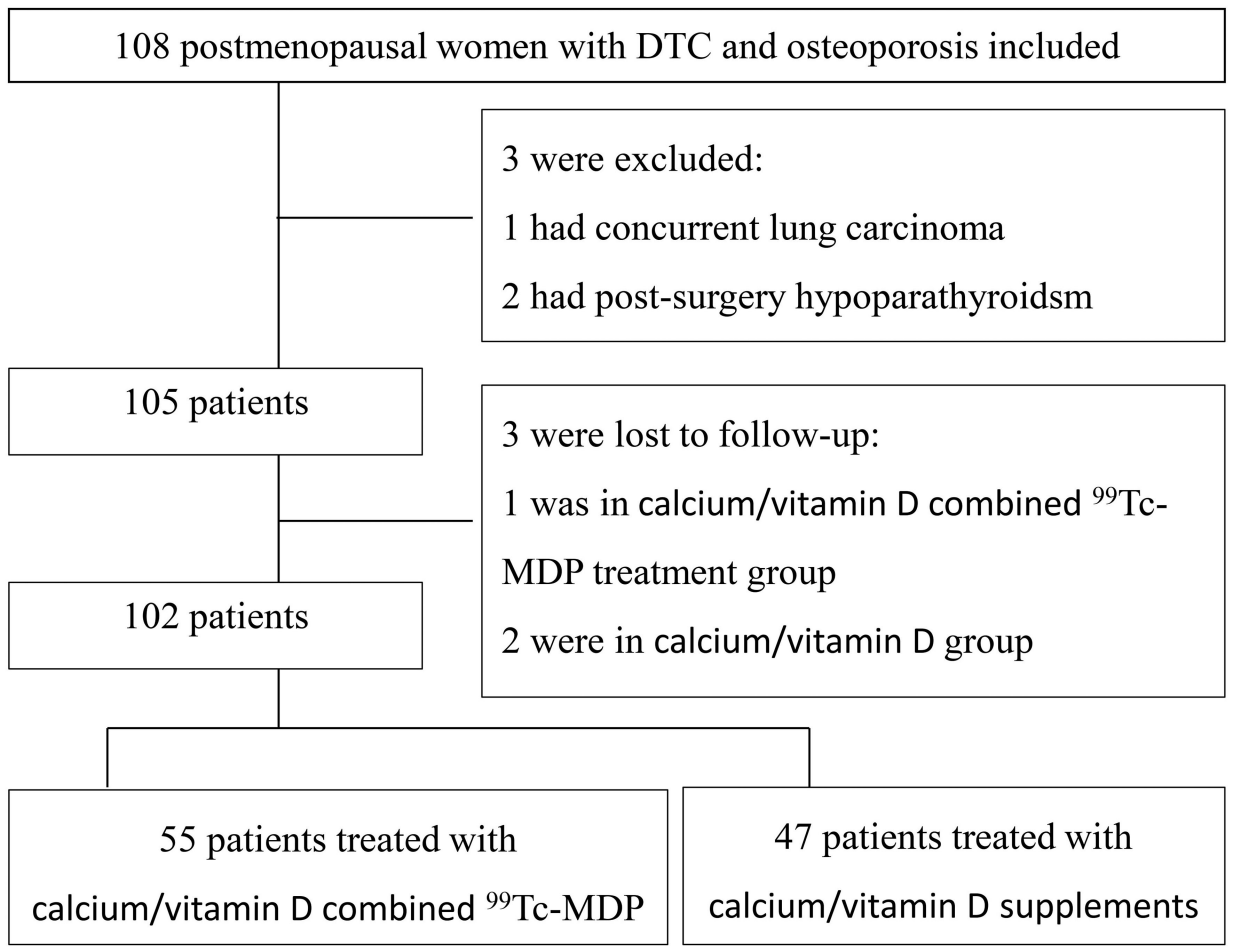
Study flow chart.

A total of 108 postmenopausal DTC patients with OP under TSH suppression therapy were enrolled. Three patients were excluded and three more were lost during follow-up. Out of the 102 included patients, 55 were in group_Ca_ and 47 were in group_mdp_ (**Figure 1**). Age, weight, BMI, TSH values, duration of TSH suppression therapy, and BMD at baseline are listed in **Table 1** and showed no significant differences (p > 0.05). In group_mdp_, 26 and 21 patients had TSH<0.1 μIU/mL and TSH between 0.1-0.5 μIU/mL, respectively, while in group_Ca_, 31 and 24 patients had TSH < 0.1 μIU/mL and TSH between 0.1–0.5 μIU/mL.

**Table 1:** Clinical characteristics of DTC patients with osteopenia.

Characteristic	^99^Tc-MDP combined treatment	Calcium and vitamin D supplements	t value	P
TSH(μIU/mL)	0.20 ± 0.16	0.22 ± 0.19	-0.63	0.53
TSH<0.1 μIU/mL	26	31		
TSH 0.1-0.5 μIU/mL	21	24		
Time of TSH suppression(months)	18.24 ± 6.88	19.38 ± 9.0	-0.54	0.59
Age(years)	61.04 ± 6.53	60.87 ± 6.67	0.12	0.91
Height (cm)	159.50 ± 4.22	158.77 ± 4.28	0.81	0.42
Weight (kg)	58.64 ± 6.53	59.67 ± 8.57	-0.68	0.50
Body mass index(kg/m^2^)	23.07 ± 2.64	23.68 ± 3.32	-1.02	0.31
C-terminal telopeptide of type I collagen (ng/mL)	0.41 ± 0.17	0.37 ± 0.18	0.84	0.4
Propeptide of type I procollagen (ng/mL)	50.57 ± 15.6	52.94 ± 18.8	-0.56	0.58
Bone mineral density at lumbar_1-4_ (g/cm^2^)	0.8903 ± 0.06	0.9148 ± 0.08	-1.13	0.258
Calcium (mmol/L)	2.19 ± 0.15	2.28 ± 0.75	-1.024	0.308
TNM (N)			Z value	P
T_1_N_1_	22	26	0.05	0.96
T_2_N_1_	12	10	0.9	0.37
T_3_N_0-1_	3	4	0.18	0.86
T_4_N_0-1_	9	13	0.55	0.58
T_x_N_1_	1	2	0.44	0.66

### Lumbar BMD

The combined ^99^Tc-MDP treatment showed a significant increase in the mean percentage change of lumbar BMD at month 12 compared with group_Ca_ (t=2.156, p=0.035), see **Table 2**. Within-group comparisons showed no significant difference in lumbar BMD in group_mdp_ at month 12 compared with baseline (t=-0.92, p= 0.38). However, a significant decrease in BMD of the lumbar spine from 0.9148 ± 0.08 to 0.8726 ± 0.08 (t=3.81, p=0.001) at month 12 was observed in group_Ca_ (**Table 2**).

**Table 2:** Bone mineral density (BMD, g/cm^2^) and mean percent change (%, $\bar{\chi }$ ± SD) of lumbar spine.

Groups	BMD (g/cm^2^) and mean percent change (%, $\bar{\chi }$ ± SD)		t value	p
	Baseline	12 months		
^99^Tc-MDP combined treatment	0.8903 ± 0.06	0.9009 ± 0.05 (1.3 ± 5%^*^)	-0.92	0.38
Calcium and vitamin D group	0.9148 ± 0.08	0.8726 ± 0.08^ϕ^ (-2.3 ± 7%)	3.81	0.001
t value	-1.13	-1.597(2.156)		
p	0.258	0.113(0.035)		

*indicates a significant difference between ^99^Tc-MDP combined treatment and the calcium/vitamin D group.

^ϕ^indicates a significant decrease in lumbar BMD at 12 months in DTC patients with osteopenia in the calcium/vitamin D treated group compared with baseline.

^99^Tc-MDP treatment showed no significant difference in the subgroup analysis of lumbar BMD in patients with a TSH level <0.1μIU/mL and between 0.1-0.5μIU/mL, The baseline lumbar BMD(g/cm^2^) and the mean percent changes (%, $\bar{\chi }$ ± SD) were 0.8943 ± 0.06 and 0.908 ± 0.07(t=0.54, p= 0.59), 0.0088 ± 0.06 and 0.1834 ± 0.05(t=0.45, p= 0.65), in patients with a suppressed TSH level <0.1μIU/mL and between 0.1-0.5μIU/mL, respectively (**Table 3**).

**Table 3:** Mean percent change (%, $\bar{\chi }$ ± SD) of lumbar spine bone mineral density and bone metabolism markers in patients with different suppressed TSH levels treated with ^99^Tc-MDP.

Groups	Bone mineral density (BMD)	Procollagen type 1 N-terminal propeptide (PINP)		β-isomer of C-terminal telopeptide of type I collagen (β-CTX)		
	Baseline (g/cm^2^) 12 months	6 months	12 months	6 months	12 months	
TSH<0.1 μIU/mL	0.8943 ± 0.06 0.885 ± 5.0	8.89 ± 16.6	26.57 ± 34.5	-2.03 ± 4.9	-3.2 ± 2.9
TSH 0.1-0.5 μIU/mL	0.908 ± 0.07 1.834 ± 5.9	9.49 ± .19.5	13.34 ± 27.3	-7.98 ± 20.5	-16.2 ± 30.4
t value	0.54 0.45	0.08	1.12	-1.08	-1.62
p	0.59 0.65	0.93	0.27	0.28	0.12

### Bone turnover markers

**Figure 2:**
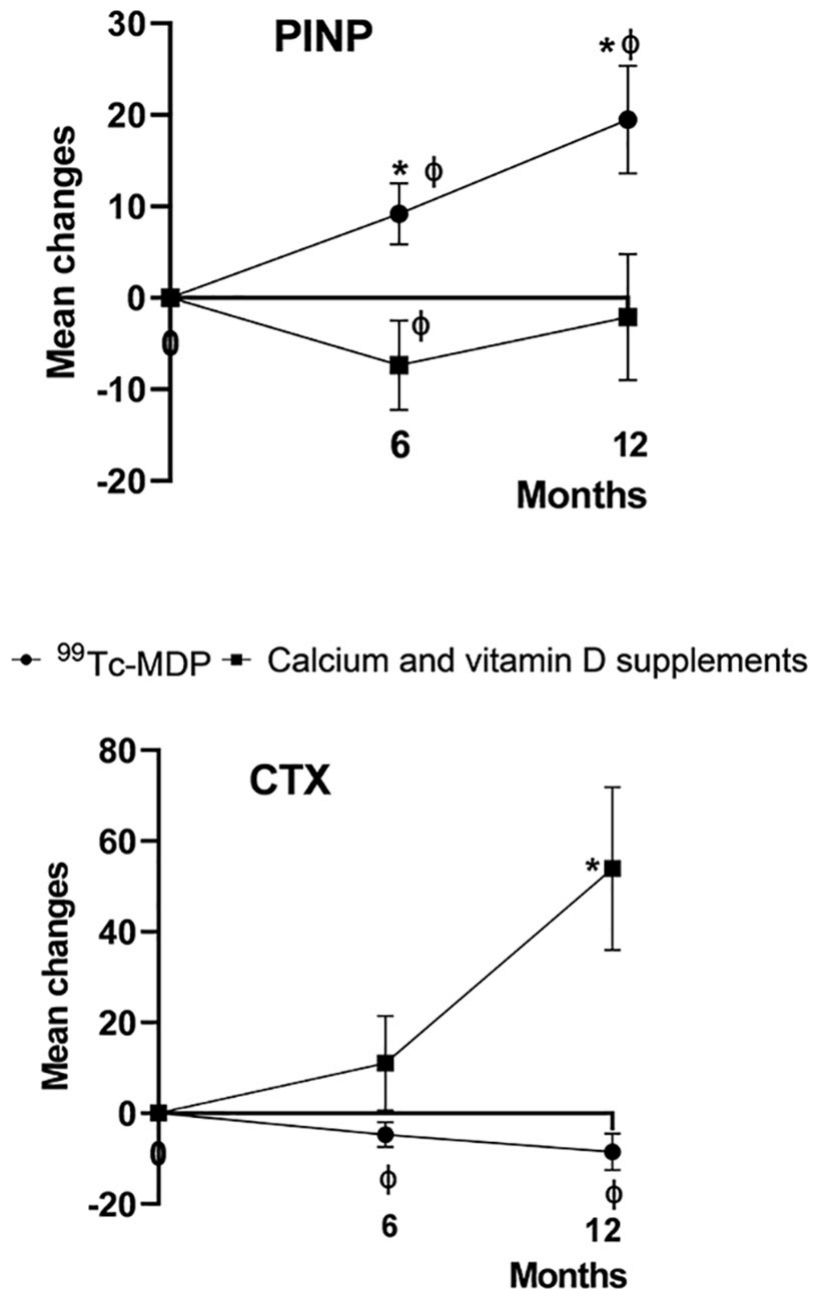
Percentage change from baseline in levels of bone turnover markers. The mean percentage change from baseline in the levels of serum β-isomer of C-terminal telopeptide of type I collagen (β-CTX) and procollagen type 1 N-terminal propeptide (P1NP) are shown at 6 and 12 months after the baseline visit. An asterisk (*) indicates p < 0.05 for comparisons between ^99^Tc-MDP combined treatment and calcium/vitamin D supplement groups. ^99^Tc-MDP combined treatment significantly increased P1NP at month 6 (t=2.37, p = 0.02) and month 12 (t =2.224, p = 0.029), and significantly decreased β-CTX at month 12 (t=-2.746, p = 0.008) compared with the calcium/vitamin D supplement group. The vertical lines represent the 95% confidence intervals at each time point. A ‘ϕ’ indicates p < 0.05 for within-group comparisons with baseline.

^99^Tc-MDP combined treatment significantly increased P1NP at month 6 (t=2.37, p = 0.02) and 12 (t=2.777, p = 0.007), and significantly decreased β-CTX at month 12 (t=-2.746, p = 0.008) compared with group_Ca_. Significant increases in P1NP (t=-2.483 and -3.12, p=0.02 and 0.004, respectively) and decreases in β-CTX (t=2.321 and 2.516, p=0.028 and 0.018, respectively) were found at months 6 and 12 in group_mdp_ compared with baseline (**Figure 2**, **Table 4**).

**Table 4:** Changes in bone metabolism markers (ng/mL) and mean percent change (%, $\bar{\chi }$ ± SD).

Groups	Procollagen type 1 N-terminal propeptide (P1NP)			β-isomer of C-terminal telopeptide of type I collagen (β-CTX)		
	Baseline	6 months	12 months	Baseline	6 months	12 months
^99^Tc-MDP combined treatment	50.57 ± 15.6	53.15 ± 14 (9.2 ± 18%*^ϕ^)	57.56 ± 14 (20.6 ± 31% ^ϕ^)	0.44 ± 0.17	0.418 ± 0.16 (-2.9 ± 11% ^ϕ^)	0.392 ± 0.16 (-8.3 ± 22%* ^ϕ^)
Calcium and vitamin D group	52.94 ± 18.8	42.26 ± 15 (-7.4 ± 33%)	47.45 ± 19 (-2.1 ± 47%)	0.35 ± 0.18	0.34 ± 0.16 (12.3 ± 68%)	0.41 ± 0.18 (60.8 ± 129%)
t value	-0.56	2.37	2.224	2.56	-1.15	-2.746
p	0.58	0.02	0.029	0.012	0.254	0.008

*indicates a significant increase in patients with ^99^Tc-MDP combined treatment compared with the calcium and vitamin D group.

^ϕ^indicates a significant increase in P1NP (t=-2.483 and -3.12, p=0.02 and 0.004, respectively), and a significant decrease in β-CTX (t=2.321 and 2.516, p=0.028 and 0.018, respectively) at months 6 and 12 in DTC patients with osteopenia treated with ^99^Tc-MDP combined treatment compared with baseline.

^99^Tc-MDP treatment showed no significant difference in the subgroup analysis of P1NP and β-CTX in patients with a TSH level <0.1μIU/mL and between 0.1-0.5μIU/mL. The mean percent changes (%, $\bar{\chi }$ ± SD) were 0.0889 ± 0.26 and 0.0949 ± 0.19 (t=0.08, p=0.93), 0.2657 ± 0.34 and 0.1334 ± 0.27 (t=1.12, p= 0.27) at months 6 and 12, respectively (**Table 3**).

### Safety

No severe adverse events were found in either group. When comparing blood counts and liver and renal function indices at baseline with those at the 12-month follow-up, no significant differences were found (p>0.05) in the group_mdp_ (**Table 5**).

**Table 5:** Changes in routine clinical chemistry parameters (p all >0.05).

Parameters	^99^Tc-MDP combined treatment		Calcium and vitamin D	
	Baseline	12 months	Baseline	12 months
Alanine aminotransferase(<52U/L)	24.4 ± 11	28.8 ± 10	26 ± 11	25.7 ± 11
Aspartate aminotransferase(<36U/L)	22.4 ± 8	26.5 ± 9	23.3 ± 6	23.9 ± 9
Urea nitrogen (2.5-6.1umol/L)	4.39 ± 1.3	4.16 ± 1.3	5.0 ± 1.6	4.84 ± 1.2
Creatinine (49-92mol/L)	56.9 ± 12	52.1 ± 9	56.3 ± 11	57.8 ± 10
White blood cells (3.5-9.5×10^9^/L)	5.27 ± 1.6	5.54 ± 1.2	6.05 ± 1.3	6.1 ± 1.4
Red blood cells (3.8-5.1×10^12^/L)	4.17 ± 0.5	4.3 ± 0.4	4.56 ± 0.3	4.67 ± 0.8
Platelet (125-135×10^9^/L)	166 ± 38	189 ± 48	175 ± 41	207 ± 51

## Discussion

DTC has become one of the most common endocrine malignancies with a good prognosis. TSH suppression treatment is necessary as tumor cells express TSH receptors on the cell membrane and respond to TSH stimulation by increasing the expression of several proteins and the rate of cell growth (3, 4, 14). However, studies, including ours, have found that excessive intake of L-T_4_ results in a negative balance of bone formation and resorption, leading to bone loss (5). Therefore, skeletal health should be an important issue in the therapeutic decision-making of patients with DTC. The 2015 ATA guidelines recommend the use of calcium and vitamin D supplementation (1) to correct the negative calcium balance induced by mild thyroid hormone excess and possibly to improve the effectiveness of bone-active agents such as bisphosphonates (15). In addition, a recent study reported that calcium plus vitamin D had important clinical significance in adjusting bone metabolism and delaying the progression of osteoporosis in patients with hyperthyroidism (16). However, the effect of calcium and vitamin D supplementation on bone loss in DTC patients with osteopenia under TSH suppression therapy remained unclear. Our results indicated that calcium plus vitamin D supplements alone cannot effectively prevent further bone loss in postmenopausal women with DTC under TSH suppression treatment. The reason may be the difference between endogenous and exogenous hyperthyroidism.

^99^Tc-MDP is a novel bisphosphonate derivative without radioactivity and has been used for osteoarthritis, necrosis of the talus, ankylosing spondylitis, and rheumatoid arthritis in China for many years (17–20). We further studied the preventive effects of ^99^Tc-MDP on bone loss in postmenopausal women with DTC under TSH suppression therapy. The study found that ^99^Tc-MDP is effective in preventing BMD loss in patients with OP. The BMD maintenance effect of ^99^Tc-MDP demonstrated in the present clinical study is, as far as we know, the first to compare outcomes with calcium/vitamin D supplements in this patient population. The possible mechanisms accounting for the improvement in BMD with ^99^Tc-MDP may include elevation of the osteogenic capacity of mesenchymal stem cells and decreased adipogenic differentiation capacity (21), induction of osteoblast proliferation and differentiation, and inhibition of osteoclast differentiation and activation by regulatory effects on the osteoprotegerin (OPG)/receptor activator of nuclear factor kappa‐B ligand (RANKL)/receptor activator of NF-κB (RANK) system (22–24). However, for patients with certain bone remodeling disorders such as osteopathia striata with cranial sclerosis (25), ^99^Tc-MDP is not recommended because its osteogenesis promoting effect. Anti-osteopenia treatment may be recommended at the diagnosis of low BMD (T-score > –2.0) and for those at risk of fractures in patients requiring TSH suppression therapy.

Reduced serum TSH levels may themselves be an individual factor associated with decreased BMD and, consequently, with a greater risk of bone fracture (26). In this study, we subdivided DTC patients into groups with suppressed TSH<0.1 level and 0.1-0.5 μIU/mL. The subgroup analysis in the ^99^Tc-MDP treatment group showed no significant differences in the mean percentage change of lumbar BMD and bone metabolism biomarkers, which may be due to the limited number of patients and the short-term follow-up. Interestingly, a systematic review and meta-analysis reported significantly higher levels of neutrophil-to-lymphocyte ratio among postmenopausal women with osteoporosis compared with postmenopausal women without osteoporosis (27). In our study, we included patients with DXA-confirmed osteopenia. Further research will be valuable for postmenopausal patients with DTC.

The potential safety issues should be considered when bisphosphonates are used (15) because nitrogen-containing bisphosphonates (N-BPs) can cause rare but serious side effects, such as atypical femoral fractures and osteonecrosis of the jaw (28–31). Intravenous zoledronic acid has been shown to induce atrial fibrillation (32, 33), and this risk may be relatively increased in patients with subclinical hyperthyroidism (34). Animal experiments (21) and our clinical study showed that ^99^Tc-MDP treatment does not cause osteonecrosis of the jaw, and no other side effects were observed.

There were several limitations to our study, such as its non-randomized nature. The effects of ^99^Tc-MDP on OP should be observed with long-term follow-up. A systematic review reported low BMD at the total hip of postmenopausal women undergoing TSH-suppressive therapy, whereas the effects on BMD at the lumbar spine and femoral neck were variable (15). Another limitation of our study was the lack of data on the total hip or femoral neck.

**Figure 3:**
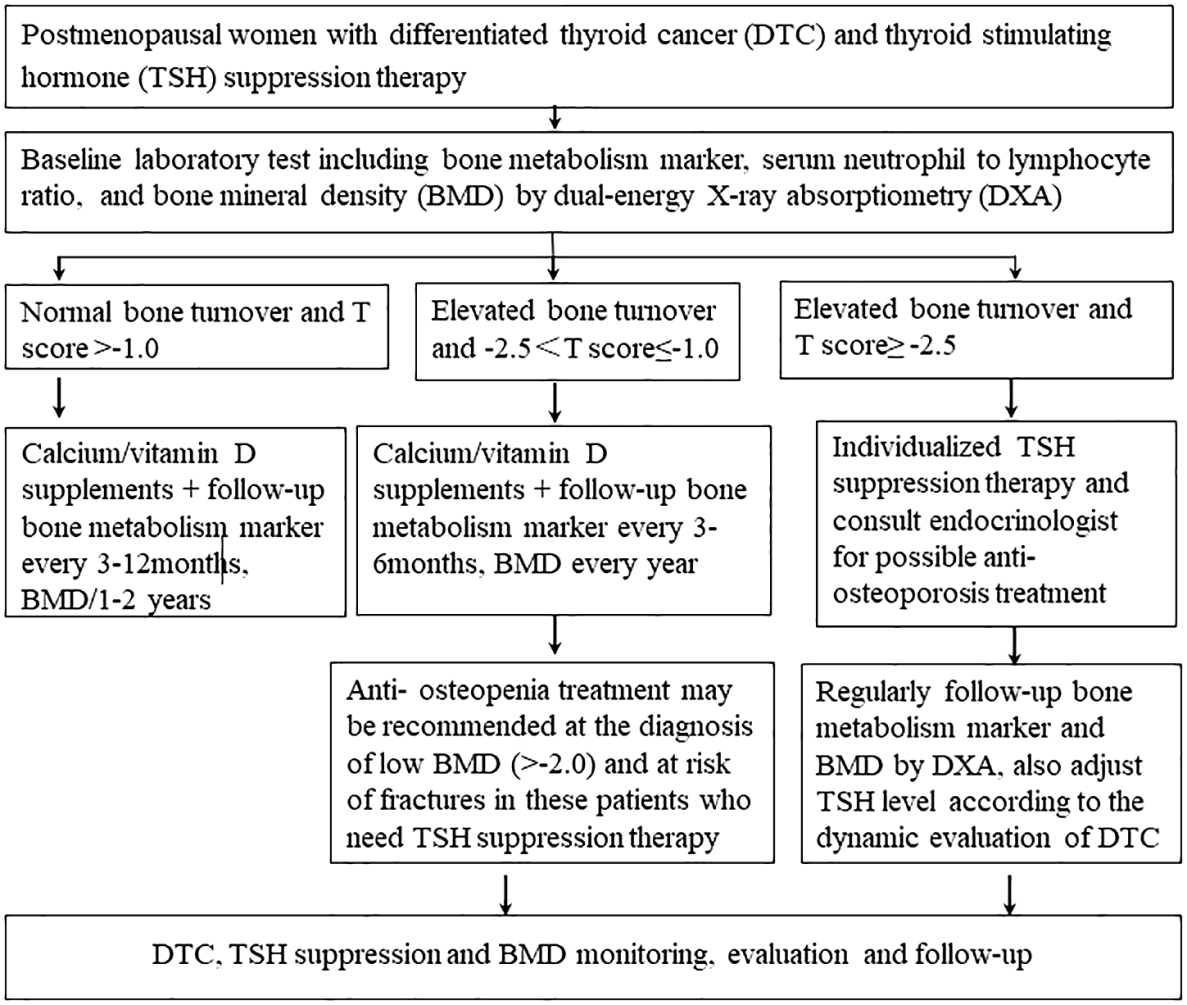
Bone health management for postmenopausal patients with differentiated thyroid cancer under thyroid-stimulating hormone suppression therapy.

In conclusion, ^99^Tc-MDP is safe and could maintain lumbar BMD in postmenopausal patients with DTC and osteopenia under TSH suppression therapy during a 1-year follow-up. Calcium/vitamin D supplementation alone could not effectively prevent bone loss in these patients. Additionally, bone health management for postmenopausal patients with DTC undergoing TSH suppression therapy is summarized in **Figure 3**.

## Data Availability

The raw data supporting the conclusions of this article will be made available by the authors, without undue reservation.
